# Anti-obesity Effect of HT048, a Herbal Combination, in High Fat Diet-Induced Obese Rats

**DOI:** 10.3390/molecules171214765

**Published:** 2012-12-11

**Authors:** Dong Wook Lim, MiKyung Song, Juyeon Park, Sang Woug Park, Nak Hoon Kim, Bhakta Prasad Gaire, Ho-Young Choi, Hocheol Kim

**Affiliations:** 1 Department of Herbal Pharmacology, College of Korean Medicine, Kyung Hee University, Seoul 130-701, Korea; 2 Korea Institute of Science and Technology for Eastern Medicine (KISTEM), NeuMed Inc., Seoul 130-701, Korea

**Keywords:** anti-obesity, herbal combination, HT048, *Citrus unshiu* peel, *Crataegus pinnatifida* fruit

## Abstract

This study evaluated the anti-obesity effects of HT048, a combination of *C. pinnatifida* fruit and *C. unshiu* peel extracts, in high-fat diet (HFD)-induced obese rats. 4-Week-old male Sprague Dawley (SD) rats were divided into normal and high fat diet (HFD) groups. The HFD groups were further divided into five groups treated with distilled water, orlistat (40 mg/kg, twice daily, p.o) and HT048 (30, 100 and 300 mg/kg, twice daily, p.o.) for 12 weeks. Orlistat, an anti-obesity drug, was used as positive control in the HFD-induced obese rats. We measured the food intake, body weight, epididymal adipose tissue and liver weights, and serum total cholesterol (TC), triglyceride (TG), alanine transaminase (ALT), and aspartate aminotransferase (AST) levels. The body weight and epididymal adipose tissue and liver weights of the HT048 100 and 300 mg/kg treated groups were significantly lower than that of the HFD control group. Also, serum TC, TG, ALT, and AST levels in the HT048 100 and 300 mg/kg treated groups were significantly decreased. Moreover, the orlistat treated group showed significantly reduced body weight and improved serum lipoprotein, compared with the HFD control group. These results show that HT048 supplements improved obesity-related body weight and serum lipoprotein parameters in a HFD-induced obese rat model.

## 1. Introduction

The World Health Organisation estimated that in 2008 more than 40 million pre-school children and 1.4 billion adults were overweight and more than half a billion adults were obese [1]. Obesity is often associated with cardiovascular risks, hypertension, dyslipidemia, and type 2 diabetes mellitus and has recognized as one of the most serious public health problems in the 21st century [2]. Among drugs used to treat obesity, rimonabant, a selective cannabinoid-1 receptor (CB 1) blocker, reduces weight by 4–5 kg on average and improves waist circumference and concentrations of HDL cholesterol and triglyceride, but an increased incidence of mood-related disorders has been reported. Sibutramine, a novel 5-HT (serotonin) and noradrenaline reuptake inhibitor (SNRI), also results in mean weight losses of 4–5 kg, but is associated with increases in blood pressure and pulse rate. Orlistat, a gastrointestinal lipase inhibitor, reduces weight by around 3 kg on average and decreases progression to diabetes in high-risk patients; adverse gastrointestinal effects are common [3]. Recently, the licenses of rimonabant and sibutramine were withdrawn due to an increased risk of psychiatric disorders and myocardial infarction or stroke, leaving only orlistat on the market [4]. However, only 15–30% of patients achieve sustained weight loss after one year of orlistat therapy [5]. Therefore, many studies have been conducted to find and develop the new anti-obesity drugs through the use of natural products that could minimize the side effects [6].

There has been increasing interest in Western medicine in the phenomenon of enhancement of effects on herbal medicines when tested in combinations, although this phenomenon is fundamental to traditional systems of herbal medicine [7]. Combinations of some compounds may synergistically increase or decrease the individual therapeutic activity or toxicity. Although, the mechanism of action of many traditional herbal medicines is still unknown, there are several instances of an herbal combination extract being more efficacious than an equivalent dose of one of its components alone [8,9,10].

HT048 is herbal formulation of *Citrus unshiu* peel and *Crataegus pinnatifida* fruit. Both of these herbs are among the most well-known traditional herbal medicines, frequently used to treat obesity in Korea. In previous studies, *Citrus* peel extracts was reported to inhibit the development of obesity in high fat diet (HFD)-induced obese mice accompanied by regulation of the mRNA expressions of genes involved in adipogenesis in the adipose tissue [11]. The hypolipidemic effect of *Citrus* peel extracts has also been studied in an *in vivo* model [12]. The major groups of bioactive components in *Citrus* peel are flavonoids, carotenoids, and phenethylamine alkaloids [13,14]. Nobiletin was reported to inhibit adipogenesis by regulating the expression of adipogenic transcription factors, including PPARγ and C/EBPα [15]. Sinensetin was reported to stimulate lipolysis in differentiated adipocytes, which is known to be stimulated by activating the AMPK signaling pathway [16]. Hesperidin has been reported to increase fecal fat excretion in obese rats via inhibition of lipase [17]. Also, β-cryptoxanthin was reported to inhibit body weight and adipocyte hypertrophy in obese mice [18]. Various *in vivo* studies have shown that the oral administration of synephrin resulted in decreased body weight gain. In humans, supplementation with 50 mg/kg synephrin increased resting metabolic rate with no effect on heart rate or blood pressure in a double blinded, randomized placebo-controlled study [19]. Previous reports showed that *C. pinnatifida* fruit extracts are capable of lowering serum TC, LDL-C and TG in hyperlipidemic humans [20]. Flavonoids, oligomeric proanthocyanidins (OPCs), and organic acids are the three major groups of bioactive components in *C. pinnatifida* fruit [21,22]. Quercetin has been shown to increase lipolysis, an effect that was synergic with resveratol [23]. Chlrogenic acid was reported to improve body weight, lipid metabolism and obesity-related hormones levels in HFD fed mice [24]. Also, OPCs have reported to increase the level of PPARα in the liver and decrease the level of PPARγ adipose tissues in hyperlipidemic rat [25]. These specific functions of each component of HT048 led us to hypothesize that HT048 could serve as a potent anti-obesity agent to remove excessive accumulation of body fat and improve blood lipid profiles by enhancing lipolysis activity in combination with the augmented degradation of fatty acids accumulation. Based on the observation, the present study was carried out to determine whether HT048 would cause an enhanced inhibition of body weight gain and serum lipoprotein levels in a HFD-induced obese rat model compared to the individual extracts. Further, the results have been compared with orlistat, an anti-obesity drug. To our knowledge, this is the first study to investigate the synergistic anti-obesity effects of combination of *C. pinnatifida fruit* and *C. unshiu* peel extracts using a HFD-induced obese rat model.

## 2. Results and Discussion

### 2.1. HPLC Chromatograms for Standardization of HT048

**Figure 1:**
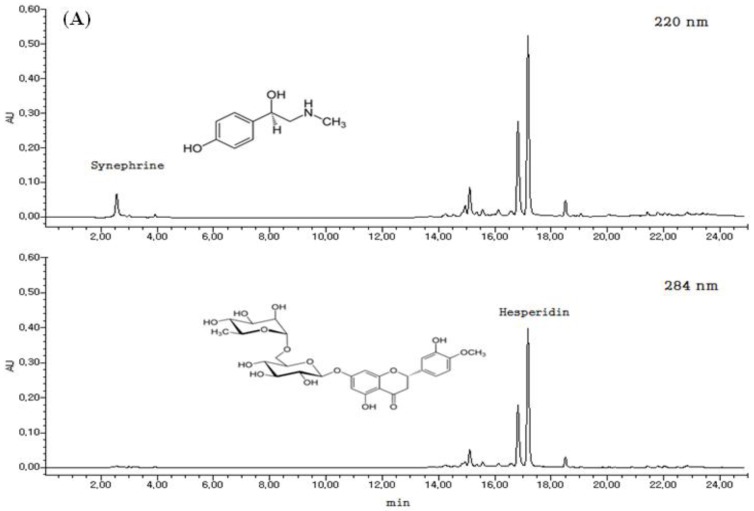

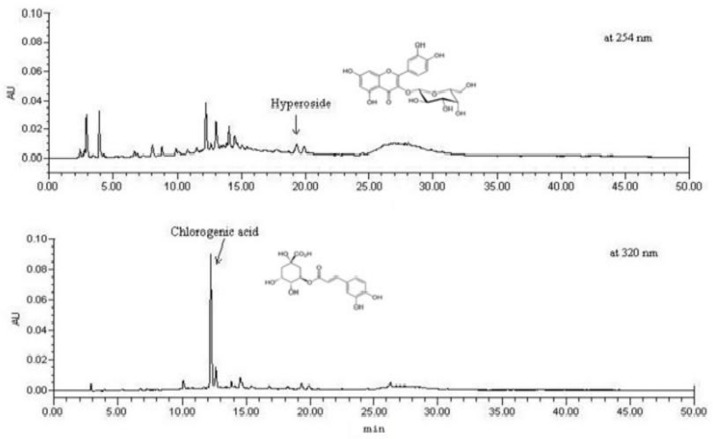
(**A**) 2-D HPLC chromatogram for standardization of *C. unshiu* peel extracts (**B**) and *C. pinnatifida* fruit extracts.

*C. unshui* peel extracts (HP425) was monitored at 220 nm for synephrine and 284 nm for hesperidin (Figure 1). The content of synephrine and hesperidin was calculated for standardization. HP425 was standardized to contain 0.5 mg/g synephrine and 21.7 mg/g hesperidin.

*C. pinnatifida* fruit extract (HP130) was monitored at 254 nm for hyperoside and 320 nm for chlorogenic acid. The content of hyperoside and chlorogenic acid was calculated for standardization. HP130 was standardized to contain 0.55 mg/g hyperoside and 1.25 mg/g chlorogenic acid. A 2-D HPLC chromatogram and the structures of the constituent compounds are shown in Figure 1.

### 2.2. Weekly Body Weight in Short-Term Treatments of HT048

**Figure 2:**
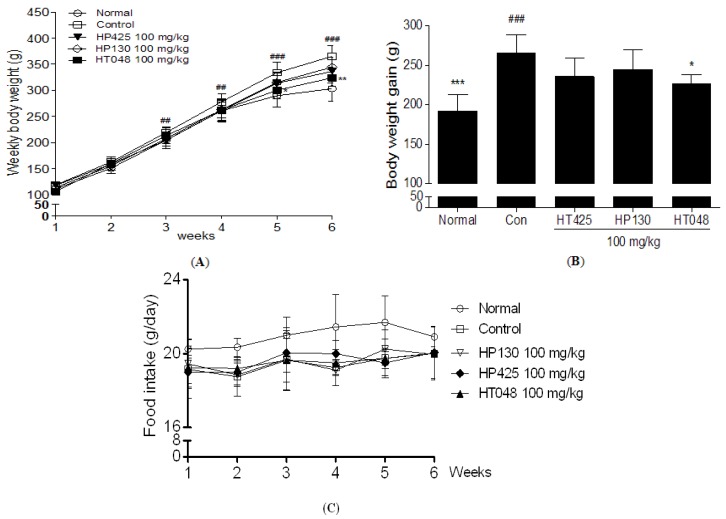
(**A**) Effects of HT048 on body weight (g), (**B**) body weight gain (g) (**C**) and food intake (g/day) in HFD induced obese rat. The body weight of the animals was recorded weekly during the experimental period. The body weight gain was calculated by the equation: final body weight—initial body weight. The food intakes were measured every day. Data are mean ± SD values (n = 10 per group). ^###^ *p* < 0.001, ^##^ *p* < 0.01, significant difference from the normal diet group. * *p* < 0.05, ** *p* < 0.01, *** *p* < 0.001, significant difference from the HFD-control group.

After six weeks on HFD, the mean body weight and body weight gain in the HFD-control group were 20.8% and 37.5% higher, respectively, than those normal group, indicating that HFD had induced obesity. A significant difference in body weight was observed between the HT048 100 mg/kg treated group and the HFD-control group by five weeks after initiating administration. After six weeks of treatments, the final mean body weight of the HT048 100 mg/kg treated group was significantly less than that of the HFD-control group (323.8 ± 15.8 g *vs.* 365 ± 20.3 g, 11.5% body weight reduction in body weight compared to the control group, *p* < 0.01, Figure 2A). Also the body weight gain of the HT048 100 mg/kg treated group was significantly less than that of the HFD-control group (228.8 ± 11.4 g *vs.* 264.9 ± 23.8 g, *p* < 0.05, Figure 2B). However, there was no significant difference in the body weight and body weight gain of the HP425 and HP130 100 mg/kg treated groups. The food intake amounts were not changed in HFD fed rats treated with HT048, HP425, and HP130 100 mg/kg (Figure 2C).

### 2.3. Weekly Body Weight in Long-Term Treatments of HT048

**Figure 3:**
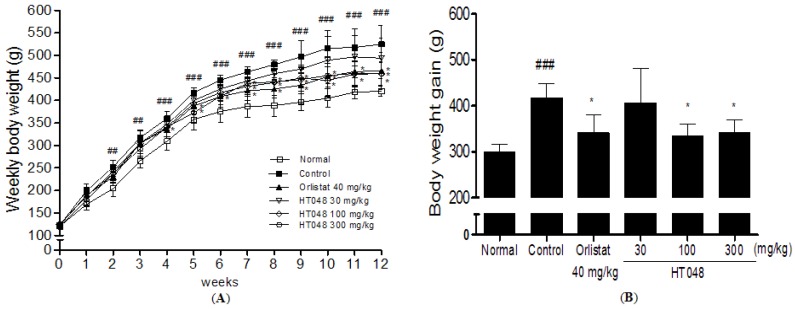

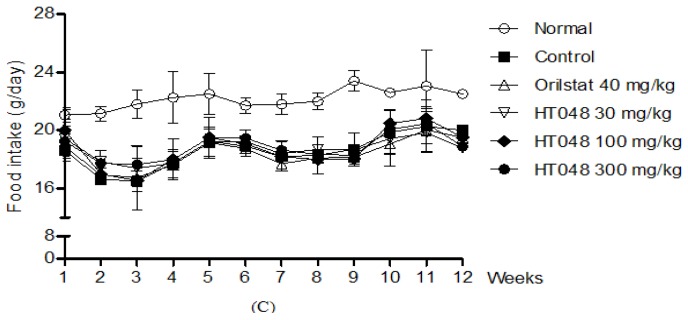
(**A**) Effects of HT048 on body weight (g), (**B**) body weight gain (g) (**C**) and food intake (g/day) in HFD induced obese rat. The body weight of the animals was recorded weekly during the experimental period. The body weight gain was calculated by the equation: final body weight—initial body weight. The food intakes were measured on a per-cage basis throughout the study every 2 or 3 days. Data are mean ± SD values (n = 10 per group). ^###^ *p* < 0.001, significant difference from the normal diet group. * *p* < 0.05, significant difference from the HFD-control group.

Body weights increased over time in all groups, but body weights increased significantly more in rats fed with HFD alone than in rats fed a normal chow diet. A significant difference in body weight was observed between the HT048 100 and 300 mg/kg treated groups and the HFD-control group by five weeks after initiating administration. After 12 weeks of treatments, the final mean body weight of the HT048 100 and 300 mg/kg treated groups were significantly less than that of the HFD-control group (459.1 ± 27.6 g *vs.* 524.8 ± 41.5 g, *p* < 0.05 and 459.3 ± 25.5 g *vs.* 524.8 ± 41.5 g, *p* < 0.05, 12.5% body weight reduction in body weight compared to the control group, Figure 3A).

Also the body weight gain of HT048 100 and 300 mg/kg treated group was significantly less than that of the HFD-control group (417.2 ± 30.4 g *vs.* 333.9 ± 26.6 g, *p* < 0.05 and 417.2 ± 30.4 g *vs.* 341.9 ± 28.0 g, *p* < 0.05) (Figure 3B). Moreover, body weight and body weight gain were significantly reduced in the orlistat 40 mg/kg treated group compared with the HFD-control group (11.4% and 18.2%, respectively). The food intake amounts were not changed in HFD fed rats treated with HT048 and orlistat (Figure 3C).

### 2.4. Epididymal Adipose Tissue and Liver Weight in Long-Term Treatments of HT048

**Figure 4:**
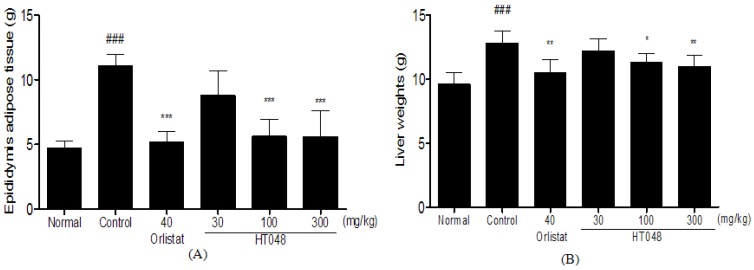
(**A**) Effects of HT048 on changes in epididymal adipose tissue (**B**) and liver weights (g). Epididymal adipose tissues and liver were dissected, washed with saline, and immediately weighted for analysis. Data are mean ± SD values (n = 10 per group). ^###^ *p* < 0.001, significant difference from the normal diet group. * *p* < 0.05, ** *p* < 0.01, *** *p* < 0.001, significant difference from the HFD-control group.

After 12 weeks on HFD, the weights of epididymal adipose tissue and liver weights were significantly higher in the HFD-control group (135.7% and 33.3%, respectively) than in the normal diet group. After 12 weeks of treatments, epididymal adipose tissue weights of the HT048 100 and 300 mg/kg treated groups were significantly less compared to the HFD-control group (49.2% and 49.5%, respectively, Figure 4A). Also, the liver weight of HT048 100 and 300 mg/kg treated groups were significantly less compared to the HFD-control group (11.7% and 14.2%, respectively, Figure 4B).

### 2.5. Serum TC, TG, ALT, and AST Concentrations in Long-Term Treatments of HT048

**Figure 5:**
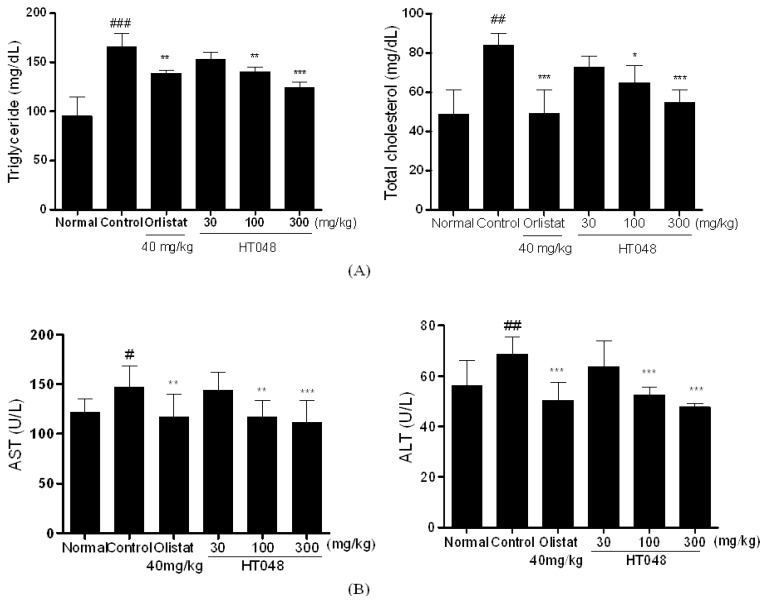
(**A**) Effects HT048 on serum triglyceride and total cholesterol, (**B**) and AST and ALT concentrations in HDF induced obese rat. At the end of the treatment period, blood sample was collected via abdominal aorta. Serum triglyceride (TG), total cholesterol (TC), ALT, and AST concentrations were measured by biochemical analyzer. Data are mean ± SD values (n = 10 per group). ^#^ *p* < 0.05, ^##^ *p* < 0.01, ^###^ *p* < 0.001, significant difference from the normal diet group. * *p* < 0.05, ** *p* < 0.01, *** *p* < 0.001, significant difference from the HFD-control group.

Serum triglyceride (TG), total cholesterol (TC), alanine transaminase (ALT), and aspartate aminotransferase (AST) levels were significantly higher in the HFD-control group compared with the normal chow diet feed group. After 12 weeks of treatments, HT048 100 and 300 mg/kg treated groups displayed significantly lower serum TG and TC concentrations compared to the HFD-control group (Figure 5A). In addition, HT048 100 and 300 mg/kg treated groups had significantly lower plasma ALT and AST concentrations compared to the HFD-control group (Figure 5B).

### 2.6. Discussion

HT048, a combination of *C. pinnatifida* fruit and *C. unshiu* peel extracts, significantly reduced body weight gain compared to the high fat diet (HFD) control group and that this was not significantly reduced in animals given *C. pinnatifida* fruit or *C. unshiu* peel extracts, however, this could have simply been because the changes in body weight in the HT048 group were less variable than in the *C. pinnatifida* fruit and *C. unshiu* peel extracts groups. HFD feeding allows the characterization of obesity development and the evaluation of anti-obesity interventions in an *in vivo* experimental model that is pathophysiologically very similar to the obese human [26]. From a nutritional perspective, a human diet with 60 kcal% fat would be considered extreme [27]. Therefore, the anti-obesity effect of HT048 was evaluated by a 60 kcal% HFD induced obesity rat model. In the present study, the rats fed the HFD for 12 weeks showed obesity, which was associated with significantly increased body weight with the development of dyslipidemia. After 12 weeks of oral administration, the body weights, and serum total cholesterol (TC) and triglyceride (TG) levels of HT048 100 and 300 mg/kg treated groups were significantly lower than those of the HFD-control group, without affecting the amount of food or energy intakes. Although measurement of food consumption was relatively crude, the results of the study indicate that chronic administration of HT048 100 and 300 mg/kg did not affect food intake. Consistent with our findings from the orlistat-treated group, the HT048 might have anti-obesity effects in HFD induced obese rat model, without affecting appetite.

Obesity is characterized by increased adipose tissue mass that results from both increased fat cell number and increased fat cell size. Adipose tissue is a dynamic organ that plays an important role in energy balance and changes in mass according to the metabolic requirements of the organism [28]. Excess energy intake and reduced energy expenditure results in abnormal excessive growth of white adipose tissue (WAT), which can lead to the development of obesity [29]. Epididymal adipose tissue in the rat is generally considered to be WAT with a characteristic structure and function [30]. After 12 weeks of oral administration, the weight of epididymal adipose tissue in HT048 100 and 300 mg/kg treated group was significantly decreased by 34.8% and 38.6% compared to the HFD-control group. These results suggest that HT048 may prevent the accumulation of WAT in HFD-induced obese rats. However, further mechanism studies are needs to clarify that the anti-obesity effects of HT048 may be elicited by regulating the expressions of lipogenesis-related genes in WAT.

We also analyzed the effects of HT048 on the development of fatty liver, which is strongly associated with obesity [31]. HFD can induce hepatic steatosis and signs of hepatic insulin resistance in the animal; this closely resembles the human obese state [32]. Xu *et al.* reported that the liver of the HFD-induced obese rodents exhibited an accumulation of numerous fatty droplets, a typical sign of fatty liver, and the liver weight was significantly higher in the HFD control group than in the normal diet group [33,34]. In our results, after 12 weeks on HFD, the liver weigh was significantly higher in the HFD-control group than in the normal diet group. Although we did not measure fat or triglyceride content of the liver, the liver weight was significantly lower in HT048 100 and 300 mg/kg treated groups than in the HFD-control group. Serum AST and ALT levels are clinically and toxicologically important indicators [35], and increase as a results of tissue damage caused by toxicants or disease conditions. In the HFD-control group, the activities of liver function markers, including serum AST and ALT, were significantly elevated relative to those in the normal diet group and were improved by HT048 supplementation. These results suggest that administration of HT048 can dramatically suppress the development of HFD-induced fatty liver.

## 3. Experimental

### 3.1. Plant Material

The dried peel of *C. unshiu* and dried *C. pinnatifida* fruit were purchased from Yaksudang Co. (Seoul, Korea). The samples were identified by Professor Hocheol Kim and voucher specimens (#HP130 and #HP425) were deposited at the Department of Herbal Pharmacology, College of Oriental Medicine, Kyung Hee University, Seoul, Korea.

### 3.2. Sample Preparation and HPLC Analysis

The dried peel of *C. unshiu* and dried *C. pinnatifida* fruit were extracted separately with 30% ethanol for 3 h at 95 °C in a reflux apparatus. The extracts were filtered and concentrated under reduced pressure, and samples were lyophilized to yield a dark yellow powder. The yield (%) of individual extracts was 23.1% and 8.4%, respectively. Then, the two kinds of powder were mixed for preparing HT048 in the proportion of the raw materials. The quantitative authentication of *C. unshui* peel and *C. pinnatifida* fruit were performed by a high performance liquid chromatography (HPLC) analysis system equipped with a Waters 1525 pump, a 2707 autosampler and a 2998 PDA detector. The chromatic separation was achieved at 30 °C on Waters Sunfire™ C_18_ (250 mm × 4 mm i.d., 5 µm particle size) column. *C. unshui* peel extracts was monitored at 220 nm for synephrine and 284 nm for hesperidin. The run time was set at 25 min and the flow rate was 1.0 mL/min and the sample injection volume was 10 μL. Mobile phases A and B were 1% H_3_PO_4_ (v/v) and CH_3_CN, respectively. Gradient elution was as follows: 0–10 min 0–40% solvent B, 10–15 min 40–100% solvent B, 15–25 min 100–0% solvent B. The content of synephrine and hesperidin was calculated for standardization. *C. unshiu* peel extract was standardized to contain 0.5 mg/g synephrine and 21.7 mg/g hesperidin. *C. pinnatifida* fruit extract was monitored at 254 nm for hyperoside and 320 nm for chlorogenic acid. The run time was set at 45 min and the flow rate was 1.0 mL/min and the sample injection volume was 10 μL. Mobile phases A and B were 0.5% H_3_PO_4_ (v/v) and CH_3_CN, respectively. Gradient elution was as follows: 0–10 min 5–20% solvent B, 10–20 min 20–20% solvent B, 20–25 min 20–40% solvent B, 25–30 min 40–30% solvent B, 30–40 min 30–30% solvent B, 40–50 min 30–5% solvent B. The content of hyperoside and chlorogenic acid was calculated for standardization. *C. pinnatifida* extract was standardized to contain 0.55 mg/g hyperoside and 1.25 mg/g chlorogenic acid.

### 3.3. Animals

Male Sprague-Dawley rats, 3 weeks old, were purchased from Samtako, Gyeonggi-do, Korea. Animals were housed at two rats per cage in an air-conditioned room at 23 ± 1 °C, 55–60% relative humidity, and a 12 h light/dark cycle (07:00 lights on, 19:00 lighs off), and were given a laboratory regular rodent diet (11.8 kcal% fat, AIN-76A, Research Diets Inc., New Brunswick, NJ, USA) for 1 week. A purified ingredient high fat diet (HFD) with 60 Kcal% fat primarily from lard (D12492, Research Diets Inc.) was used to induce a rapid increase in body weight and obesity. The caloric density of the normal diet (ND) was 3.8 kcal/g and that of HFD was 5.1 kcal/g, resulting in lower daily food consumption in grams for rats fed the HFD. All of animal experiments were carried out according to the guidelines of the Kyung Hee University’s Institutional Animal Care and Use Committee (KHUASP (SE)-11-013).

### 3.4. Short-Term Treatments of HT048

After acclimatization for 1 week, 4-week-old male SD rats were randomly divided into five groups (n = 10 per group), and respectively fed a normal diet (ND) + vehicle, a high-fat diet (HFD), + vehicle, HFD + *C. pinnatifida* fruit 100 mg/kg, HFD + *C. unshiu* peel 100 mg/kg, and HFD + HT048 100 mg/kg. HT048, *C. pinnatifida* fruit and *C. unshiu* peel were dissolved in distilled water for oral administration at the desired doses in a volume of 5 mL/kg twice daily at 8:00 and 20:00 hours. All groups were treated for 6 weeks. The animals’ body weights were measured daily, and food intake was measured on a per-cage basis throughout the study every 2 or 3 days. Food intake (g/rat/day) was determined by subtracting the remaining food weight from the initial food weight of the previous feeding day and dividing by the number of rat housed in the cage.

### 3.5. Long-Term Treatments of HT048

For long term treatments with HT048, after acclimatization for 1 week, 4-week-old male SD rats were randomly divided into six groups (n = 10 per group), and respectively fed a normal diet (ND) + vehicle, a high-fat diet (HFD, 60 kcal% fat based on AIN76) + vehicle, HFD + orlistat 40 mg/kg, HFD + HT048 30 mg/kg, HFD + HT048 100 mg/kg, and HFD + HT048 300 mg/kg. The doses of HT048 were determined by preliminary tests using concentrations of 30, 100, and 300 mg/kg for selection of anti-obesity effect. HT048 was dissolved in distilled water for oral administration at the desired doses in a volume of 5 mL/kg twice daily at 8:00 and 20:00 hours. Orlistat (Xenical^®^, Roche, Basel, Switzerland) dissolved in distilled water, with 1% dimethyl sulfoxide (DMSO) and 0.1% Tween 20. All groups were treated for 12 weeks. All groups were treated for 12 weeks. The animals’ body weights were measured daily, and the food intakes (g/rat/day) were measured every 2 or 3 days. At the end of the treatment period, the rats were fasted for 12 h, and blood was collected via abdominal aorta. Liver and epididymal adipose tissues were dissected, washed with saline solution, and immediately weighted for analysis.

### 3.6. Biochemical Serum Analyses

The serum samples were prepared by centrifugation of the collected blood samples (2,500 rpm for 15 min), then stored at −80 °C for biochemical determinations. Serum triglyceride (TG), total cholesterol (TC), alanine transaminase (ALT), and aspartate aminotransferase (AST) concentrations were measured by VetTest 8008 (IDEXX Lab Inc., Westbrook, ME, USA).

### 3.7. Statistical Analysis

All data were presented as the mean ± standard deviation (SD). The effects of different treatments were compared by the ANOVA test for multiple comparisons using GraphPad Prism 5 (GraphPad Software Inc., La Jolla, San Diego, CA, USA). *p* < 0.05 was considered statistically significant.

## 4. Conclusions

In conclusion, HT048, a combination of *C. pinnatifida* fruit and *C. unshiu* peel extracts, may prevent body weight increases and improve dyslipidemia in high fat diet-induced obese rats. However, further detailed mechanism investigation of the anti-obesity effects of HT048 on lipid metabolism is required.
